# Low sex hormone-binding globulin is associated with hypertension: a cross-sectional study in a Swedish population

**DOI:** 10.1186/1471-2261-13-30

**Published:** 2013-04-18

**Authors:** Bledar Daka, Thord Rosen, Per Anders Jansson, Charlotte A Larsson, Lennart Råstam, Ulf Lindblad

**Affiliations:** 1 Department of Primary Health Care, Institute of Medicine, Gothenburg, Sweden; 2 Department of Endocrinology, Gothenburg, Sweden; 3 Department of Internal Medicine, University of Gothenburg, PO Box 454, Gothenburg SE-405 30, Sweden; 4 Department of Clinical Sciences, Malmö, Social Medicine and Global Health, CRC 28-12, Lund University, Jan Waldenströms gata 35, Malmö 205 02, Sweden

**Keywords:** Sex hormone binding globulin (SHBG), Testosterone, Gender, Hypertension, BMI

## Abstract

**Background:**

The aim of this study was to investigate the association of sex hormone-binding globulin (SHBG) and hypertension in a Swedish population.

**Methods:**

The study is based on a random sample of a Swedish population of men and women aged 30–74 years (n=2,816). Total testosterone, oestradiol and SHBG were measured in 2,782 participants. Free androgen index was then calculated according to the formula FAI=100 × (Total testosterone)/SHBG. Hypertension was diagnosed according to JNC7.

**Results:**

In men, but not in women, significant association between SHBG and both diastolic (diastolic blood pressure: β=−0.143 p<0.001) and systolic blood pressure (systolic blood pressure β=−0.114 p<0.001) was found. The association was still significant after adjusting for age, body mass index (BMI), homeostatic model assessment insulin resistance (HOMA-IR), triglycerides, high density lipoproteins (HDL) and C-reactive protein (CRP) (diastolic blood pressure: β=−0.113 p<0.001; systolic blood pressure β=−0.093 p=0.001). An inverse association was observed between SHBG and hypertension in both men (B=−0.024 p<0.001) and women (B=−0.022 p<0.001). The association was still significant in women older than 50 years after adjustments for age, BMI, physical activity, CRP and alcohol consumption (B=−0.014, p=0.008).

**Conclusion:**

In conclusion, these results show a strong association between SHBG and blood pressure independent of major determinants of high blood pressure. This association might be addressed to direct effects of SHBG in endothelial cells through the receptor for SHBG. If this is confirmed by other observational and experimental studies, it might become a new field for the development of therapies for lowering blood pressure.

## Background

Low concentrations of total testosterone in men have previously been associated with obesity, diabetes mellitus and hypertension, independent of age [1-6]. Studies in male rabbit models have shown that castration increases atherosclerosis [7]. The effects of testosterone on blood pressure levels may be explained by at least three factors. Firstly, testosterone is a vasodilatator *in vitro*, and *in vivo* experiments show a direct vasodilatation effect of testosterone [8]. Secondly, low concentrations of testosterone in men have been associated with higher total cholesterol and LDL-cholesterol and to lower levels of HDL-cholesterol, and consequently to atherosclerosis and hypertension [5,9-13]. Thirdly, an increased inflammatory response is associated with endothelial dysfunction and to an increased arterial stiffness and hypertension. Several clinical trials of testosterone replacement therapy in men with testosterone deficiency have shown a decrease in pro-inflammatory cytokines [14,15], indicating an immune-modulatory effect of testosterone. Nevertheless, the use of anabolic steroids (synthetic derivate of testosterone) is associated with hypertension [16].

A sizeable fraction of circulating testosterone is bound to sex hormone-binding globulin (SHBG); however, it is still not established if the observed link between the total testosterone and several cardiovascular risk factors (atherogenic lipid profile, type 2 diabetes, obesity) mirrors an effect of free circulating testosterone, or whether SHBG plays a more direct role. A recent study by Ding et al. [17] showed a strong association of SHBG as well as genes controlling the expression of SHBG with the risk for type 2 diabetes, but not between free testosterone and type 2 diabetes. While these results are supported by other authors [18], the role of SHBG in the development of type 2 diabetes is still subject to discussions and its contribution to the development of hypertension has to our knowledge not been investigated at all.

The purpose of this study was therefore to investigate the association between hypertension and SHBG, in both men and women in a Swedish population.

## Methods

Between 2001 and 2005 a random sample of subjects, aged 30–74 years residing in two municipalities in South-western Sweden, were enrolled in the study [19]. All individuals that responded to the study invitation and gave written and informed consent were included as participants if they also contributed with physical examination, filling in the questionnaire, and by donating venous blood. Participants were stratified by gender in 5-year age groups. In Vara, 1,811 participants were enrolled (81% participation rate), while the survey in Skövde included 1,005 subjects (70%), with the overall participation rate being 76%. After exclusion of subjects without successful analyses for SHBG, 2,782 subjects (Men=1,385; Women=1,397) remained to be analysed.

### Medical history, socio-economic and life style factors

Details on the study protocol have been published previously [19]. Standard questionnaires were used to gain information on previous hospitalizations, medication (including preventive hormonal medication and post menopausal hormonal medication), smoking and alcohol habits as well as leisure time physical activity (LTPA). Standard instruments were also used for the collection of data on demographic and socio-economic factors such as social networks, social stress, and symptoms of anxiety and depression.

LTPA was characterised based on the response of questionnaire item: ‘How much physical activity do you engage in during your leisure time?’ [20] The question referred to the past year and the answer alternatives were: 1. Sedentary leisure time: Reading, TV, stamp collecting or other sedentary activity; 2. Light LTPA: Walking, cycling or other physical activity under at least four hours per week; 3. Moderate LTPA: Running, swimming, tennis, aerobic, heavier gardening or similar physical activity during at least 2 hours a week; and 4. Heavy training or competitive sport: Heavy training or competitions in running, skiing, swimming, football, etc. performed regularly and several times per week. Smoking habits was defined as current, former or daily smoking. Alcohol consumption was assessed by a standardized set of questions on how many days during the last 30 days had the subjects consumed beer, wine and strong liquor, respectively. Each of these questions was followed by questions on how many cans, glasses and/or bottles were normally consumed on such days. The total gram of alcohol consumed per week was then calculated by multiplying the number of days of alcohol drinking with the gram of alcohol that the items of consumed alcoholic beverage contain [21].

### Physical examination

The nurses measured the standard resting blood pressure twice, one minute apart after five minutes rest with the subjects in a supine position. (Right brachial artery, arm at heart level, cuff size adjusted for arm circumference and reading the pressure at the closest 2 mmHg). The mean value of the two blood pressure readings was used for the analyses.

### Clinical chemistry

Samples including plasma and serum were drawn after an overnight fasting and were immediately frozen at −82°C. In participants without known diabetes mellitus (DM), an OGTT was performed. Total cholesterol, HDL-cholesterol, LDL-cholesterol and S-triglycerides were analysed using standard procedures.

Total testosterone was analysed with kits Beckman Coulter 2003, 386982A (CV=7-8%) and estradiol (CV=9-15%) and sex hormone-binding globulin (SHBG) (CV=5%) were analysed by kits Siemens Immulite 2000XPi [22]. These analyses were conducted in Malmö (Skåne University Hospital) and at Unilabs at Skaraborg Hospital in Skövde with results expressed in nmol/L. In 19 cases, the SHBG concentrations were reported to be higher than 180 nmol/L; however, they were not further specified. Thus, we assumed those to be 180 nmol/L. We calculated a free androgen index (FAI) for the estimation of free testosterone by the formula FAI=100xTotal testosterone/SHBG [23].

### Phenotypic characterisation

Impaired fasting glucose, impaired glucose tolerance and diabetes mellitus were defined according to the WHO criteria [24]. Hypertension was defined according to the JNC 7 criteria [25]. BMI was calculated using the formula BMI=weight(kg)/high(m)^2^. Insulin resistance was estimated using the homeostatic model assessment for insulin resistance (HOMA-IR) [26].

### Ethical considerations

All participants gave signed informed consent before being enrolled in the study, and the Ethical Committee at the University of Gothenburg, Sweden, approved the study.

### Statistics

Standard methods were used for descriptive statistics. The association between SHBG, sex hormones and blood pressure was investigated in simple and multiple linear regression models after exclusion of subjects treated with blood pressure lowering drugs. Logistic regression analyses were performed to estimate the association between hypertension and sex hormones-SHBG. Associations were expressed as regression coefficients (B) and odds ratios (OR), respectively, both with 95% confidence intervals (CI). General linear models were used to estimate differences (CI) between continuous variables. Theoretical multivariate models were used to estimate the role of possible confounders on the investigated association. All analyses were two-sided and the significance was accepted if p<0.05. All analyses were performed using SPSS Statistics for Mac. In order to evaluate the strength of the association for each possible risk factors of hypertension, we standardised the variables in consideration by the formula SV=V/SD, where SV is the standardised variable. V is the value of the variable and SD is the standard deviation. We then ranked their association with hypertension by the regression coefficient β in the logistic regression analyses.

## Results

The characteristics of the study population are presented in Table 1. In men, the SHBG increased linearly with age but in women there was a u-shaped association between age and SHBG. A strong negative association between SHBG and BMI, fasting plasma glucose, HOMA-IR and TG was found in both men and women and the same was true for systolic and diastolic blood pressure. SHBG was strongly associated with total testosterone in men and oestradiol in women.

**Table 1:** Phenotypical characterisation of sex hormone-binding globulin in a Swedish population of men and women

	**Men 1,385**						**Women 1,397**					
		***Quartiles for SHBG***				***p-trend***		***Quartiles for SHBG***				***p-trend***
	***All***	**Q1**	**Q2**	**Q3**	**Q4**		***All***	**Q1**	**Q2**	**Q3**	**Q4**	
SHBG	33±14	17.8	26.7	35.1	50.8		55±31	26.2	41.1	55.6	92.6	
Age	47.8±11.8	42.2	45.6	48.8	54.4	<0.001	47.7±11.7	46.9	47.3	49.0	47.7	0.033
BMI	26.9±3.6	28.9	27.3	26.5	24.9	<0.001	26.8±5.3	30.1	27.2	26.5	24.9	<0.001
Fasting insulin	6.7±5.2	8.9	7.0	6.2	4.9	<0.001	6.2±4.4	8.5	6.2	5.1	4.7	<0.001
Fasting glucose	5.5±1.1	5.8	5.7	5.5	5.4	<0.001	5.3±1.1	5.7	5.4	5.1	5.1	<0.001
HOMA-IR	1.7±1.6	2.3	1.8	1.5	1.1	<0.001	1.5±1.3	2.2	1.5	1.2	1.1	<0.001
Hs-CRP	2.4±5.8	3.0	2.3	1.9	3.3	0.038	2.7±4.6	4.1	2.5	2.2	2.3	0.097
LDL	3.4±0.9	3.4	3.4	3.4	3.3	0.327	3.1±0.9	3.2	3.1	3.2	3.2	0.287
HDL	1.2±0.3	1.1	1.2	1.2	1.3	<0.001	1.4±0.3	1.2	1.4	1.5	1.5	<0.001
Triglycerides	1.5±0.9	1.9	1.5	1.3	1.1	<0.001	1.2±0.6	1.4	1.1	1.0	1.1	0.004
Systolic BP	123±16	126	124	123	123	<0.001	119±18	123	118	117	119	0.008
Diastolic BP	72±10	73.8	71.7	71.8	70.6	<0.001	69±10	70.0	68.4	67.8	68.2	0.007
Total Testosterone	14.3±4.4	11.2	13.4	15.2	17.5	<0.001	1.3±1.3	1.3	1.2	1.2	1.3	0.853
Oestradiol	127±56	125	128	124	132	0.026	320±396	255	328	337	363	<0.001
FAI	48.5±17.3	64.5	50.5	43.4	35.3	<0.001	3.1±5.8	5.7	2.9	2.4	1.4	<0.001

SHBG= sex hormone-binding globulin, Q1 is the lowest quartile and Q4 is the highest. BMI=Body mass index, HOMA-IR= homeostatic model assessment insulin resistance, hs-CRP=high sensitive c-reactive protein, LDL=low density lipoprotein, HDL=high density protein, BP= blood pressure, FAI=free androgen index.

General linear models to estimate mean values and differences between quartiles were used. The significance was estimated with p-value.

In our population 271 (10%) individuals were under treatment for hypertension. The association between SHBG and blood pressure after excluding subjects on medication for hypertension is presented in Table 2. In men, significant association between SHBG and both diastolic (diastolic blood pressure: β=−0.143 p<0.001) and systolic blood pressure (systolic blood pressure β=−0.114 p<0.001) was found. The association was still significant after adjusting for age, BMI, HOMA-IR, triglycerides, HDL and CRP (diastolic blood pressure: β=−0.113 p<0.001; systolic blood pressure β=−0.093 p=0.001). In women, no association between SHBG and systolic and diastolic blood pressure, respectively, was found.

**Table 2:** The association between total testosterone, SHBG, oestradiol, free testosterone, respectively, and blood pressure in men and women

	**Men 1,255**				**Women 1,259**			
	**Systolic blood pressure**		**Diastolic blood pressure**		**Systolic blood pressure**		**Diastolic blood pressure**	
	**β**	**p**	**β**	**P**	**β**	**p**	**β**	**P**
***Model 1 Adjusted for age***								
Total testosterone	-0.136	<0.001	-0.121	<0.001	-0.011	0.470	-0.013	0.638
Sex hormone-binding globulin	-0.114	<0.001	-0.143	<0.001	-0.031	0.193	-0.055	0.041
Oestradiol	-0.014	0.560	0.022	0.408	-0.035	0.144	-0.057	0.036
Free testosterone	-0.051	0.119	-0.051	0.154	0.009	0.698	0.016	0.574
***Model 2 Adjusted for age and BMI***								
Total testosterone	-0.114	<0.001	-0.098	0.001	-0.008	0.873	-0.018	0.576
Sex hormone-binding globulin	-0.083	0.005	-0.117	<0.001	0.020	0.685	-0.018	0.610
Oestradiol	-0.019	0.440	0.017	0.530	-0.036	0.121	-0.025	0.461
Free testosterone	-0.039	0.243	-0.035	0.328	-0.016	0.570	-0.001	0.988
***Model 3 Adjusted for age, BMI, TG, HDL and CRP***								
Total testosterone	-0.111	<0.001	-0.089	0.002	-0.002	0.932	-0.014	0.468
Sex hormone-binding globulin	-0.078	0.009	-0.105	0.001	0.043	0.146	0.004	0.908
Oestradiol	-0.026	0.295	0.000	0.999	-0.008	0.768	-0.020	0.556
Free testosterone	-0.034	0.348	-0.033	0.363	-0.019	0.487	-0.006	0.855
***Model 4 Adjusted for age, HOMA-IR, Tg, HDL and CRP***								
Total testosterone	-0.123	<0.001	-0.097	0.001	0.005	0.846	-0.008	0.814
Sex hormone-binding globulin	-0.093	0.001	-0.113	<0.001	0.041	0.166	0.013	0.718
Oestradiol	-0.028	0.277	0.000	0.994	-0.004	0.902	-0.017	0.611
Free testosterone	-0.035	0.307	-0.032	0.878	-0.014	0.620	-0.005	0.876

BMI: Body mass index, Tg: triglycerides, HOMA-IR: homeostatic model assessment insulin resistance, HDL: high density lipoprotein, CRP: c-reactive protein, β: regressions coefficient, p: p value. Linear regression analysis is used to investigate the association between hormonal levels and blood pressure. All subjects with known hypertension (271) were excluded from the analyses.

**Figure 1:**
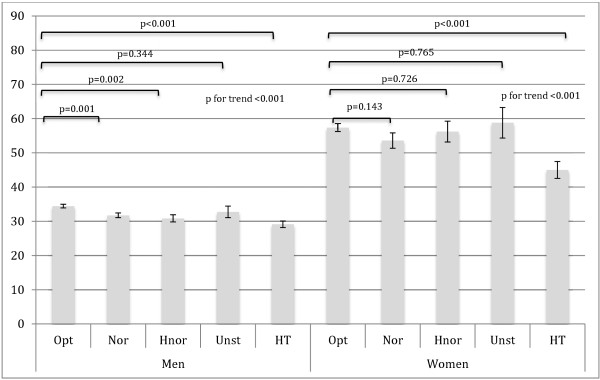
**Comparison of serum concentrations of sex hormone-binding globulin (y-axis) between different blood pressure categories.** JNC7 blood pressure categories; Opt=normal optimal BP <120/80 mm Hg, Nor= normal BP <130/85 mm Hg, Hnor= normal high BP <140/90 mm Hg, Unst= unstable blood pressure Hypertension was defined as known documented diagnosis for high blood pressure, or by three consecutive BP reading ≥140/90 mm Hg (systolic and/or diastolic). When the BP exceeded these limits only once or twice the BP was categorized as unstable.

Age-adjusted differences of SHBG concentrations in different blood pressure categories in accordance with JNC7 are presented in Figure 1. In men, SHBG decreased significantly with the severity of high blood pressure. In women, however, the SHBG was almost constant in categories NT and prehypertension1-3. Hypertensive women had significantly lower concentrations of SHBG when compared with all other categories (p<0.001).

The associations between quartiles of SHBG and hypertension are presented in Table 3 for both men and women. As observed, in women the odds for hypertension were higher for the lowest quartile (OR=4.2 p<0.001). The association was different when comparing women under or over age 50 years (Women under 50 OR=2.8, CI 1.14-7.22, p=0.025, women over 50 OR=4.8, CI 2.61-8.88, p<0.001). In women under 50, the association was not significant when adjusting for age and BMI. In contrary, for women over 50, the association remained significant when we adjusted for age, HOMA-IR, smoking habits, hs-CRP, LDL, Tg, hormone-replacement therapy and alcohol consumption (p-trend=0.05). The association remained significant even when we included a known history of stroke and diabetes in the equation (p-trend=0,043). In men, an association between quartiles of SHBG and hypertension (OR=2.2 p=0.007) was found. The association was not significant when adjusting for BMI (Table 3).

**Table 3:** Multivariate analyses of OR for hypertension associated with quartiles of sex hormone-binding globulin in women and men, respectively

**Men**		**OR**	**CI**	**P**	***Women***	**OR**	**CI**	**P**
*Model 1 Age adjusted OR for HYPERTENSION*								
All	Q 1 vs 4	2.2	1.24-3.75	0.007	All	4.2	2.45-7.06	<0.001
	Q 2 vs 4	1.7	1.05-2.78	0.031		2.3	1.31-3.91	0.004
	Q 3 vs 4	1.2	0.72-1.82	0.549		1.9	1.12-3.26	0.017
<50 y	Q 1 vs 4	1.9	0.76-4.68	0.174	<50 y	2.8	1.14-7.22	0.025
	Q 2 vs 4	1.6	0.61-4.24	0.333		1.7	0.61-4.49	0.317
	Q 3 vs 4	0.7	0.25-2.18	0.582		1.6	0.53-4.98	0.403
>50 y	Q 1 vs 4	2.1	0.99-4.66	0.053	≥50 y	4.8	2.61-8.88	<0.001
	Q 2 vs 4	1.6	0.92-2.78	0.095		2.3	1.21-4.22	0.010
	Q 3 vs 4	1.2	0.70-1.95	0.546		2.1	1.17-3.82	0.013
*Model 2 Age and BMI adjusted OR for hypertension*								
All	Q 1 vs 4	1.5	0.82-2.67	0.193	≥50 y	3.1	1.54-6.04	0.001
	Q 2 vs 4	1.3	0.81-2.22	0.250		1.9	1.00-3.79	0.049
	Q 3 vs 4	1.0	0.63-1.61	0.982		2.2	1.20-4.15	0.012
*Model 3 Age, BMI, smoking habits, hs-CRP, LDL, triglycerides, and alcohol consumption adjusted*								
All	Q 1 vs 4	1.5	0.78-2.70	0.235	≥50 y	3.0	1.47-6.33	0.003
	Q 2 vs 4	1.4	0.81-2.22	0.217		2.1	1.04-4.09	0.038
	Q 3 vs 4	1.0	0.59-1.57	0.867		2.1	1.12-4.04	0.021
*Model 4 Age, HOMA-IR, smoking habits, hs-CRP, LDL, triglycerides, and alcohol consumption adjusted*								
All	Q 1 vs 4	1.8	0.96-3.27	0.067	≥50 y	2.7	1.33-5.60	0.006
	Q 2 vs 4	1.4	0.83-2.43	0.202		1.9	0.95-3.67	0.070
	Q 3 vs 4	1.1	0.67-1.79	0.721		1.8	0.97-3.41	0.061

BMI: Body mass index, Tg: triglycerides, HOMA-IR: homeostatic model assessment insulin resistance, LDL: Low density lipoprotein, CRP: c-reactive protein, HRT: hormone replacement therapy, OR: odds ration for hypertension, p: p value. Logistic regression analysis is used to investigate the association between hormonal levels and hypertension. Possible confounders were taken into consideration in different models stepwise.

In Table 4 we ranked age, HOMA-IR, BMI and SHBG by regression coefficient in order to evaluate the strength of the association with hypertension. Age was strongest associated with hypertension and BMI was second in both men and women. The association between SHBG and hypertension was stronger than the association between HOMA-IR (surrogate of insulin resistance) and hypertension in men but not in women.

**Table 4:** Association between hypertension and sex hormone-binding globulin when major risk factors for hypertension are considered

**Men**			**β**	**p**	**Exp**	**Women**	**β**	**p**	**Exp**
	All	*Age*	1.352	<0.001	3.9		1.299	<0.001	3.7
		*BMI*	0.298	0.004	1.3		0.376	0.002	1.5
		*HOMA-IR*	0.060	0.497	1.1		0.347	0.002	1.4
		*SHBG*	-0.194	0.061	0.8		-0.297	0.027	0.7
	<50	*Age*	0.619	<0.001	1.9		0.967	<0.001	2.6
		*BMI*	0.298	0.004	1.3		0.532	0.012	1.7
		*HOMA-IR*	0.049	0.497	1.1		0.018	0.927	1.0
		*SHBG*	-0.164	0.061	0.8		-0.103	0.698	0.9
	≥50	*Age*	0.882	<0.001	2.4		0.723	<0.001	2.1
		*BMI*	0.298	0.004	1.3		0.382	0.012	1.5
		*HOMA-IR*	0.072	0.491	1.1		0.538	0.001	1.7
		*SHBG*	-0.210	0.063	0.8		-0.333	0.033	0.7

BMI: Body mass index, HOMA-IR: homeostatic model assessment insulin resistance, SHBG: sex hormone-binding globulin, β: regressions coefficient, p: p value, Exp: the odds change for hypertension for increasing with 1 standard deviation of variable. Age, BMI, HOMA-IR are standardised in order to compare the impact in hypertension. Logistic regression analysis is used to rank the strength of the association. All women with hormonal therapy are excluded from the analyses. Every considered variable is standardised. The purpose was to rank the strength of the association with hypertension for each variable and the value of β decide the strength of the association. We standardised the variables in consideration by the formula SV=V/SD, where SV is the standardised variable. V is the value of the variable and SD is the standard deviation.

The interaction between sex hormones, SHBG and hypertension was investigated in a logistic regression model, but no significant interaction was found. The interaction between age and SHBG in the hypertension and blood pressure was also investigated with regression models, but no significant difference was found.

## Discussion

In this cross-sectional study, a strong inverse association between blood pressure and SHBG was observed. Post-menopausal women with hypertension had significantly lower SHBG concentrations, and this association was significant even after adjusting for major risk factors for hypertension such as age, BMI, diabetes and insulin resistance. In men, we observed an inverse association between systolic blood pressure and SHBG. This association was independent of major confounders. Both these findings indicate that low concentrations of SHBG may have independent negative effects in the control of blood pressure.

To our knowledge this is the first study to estimate the specific role of SHBG in blood pressure control. There are some publications concerning the association between hypertension and SHBG in men, but very few in women. The majority of studies, aimed to estimate the effects of testosterone on blood pressure in men, provided information about the level of SHBG. This was done in order to estimate the concentrations of free testosterone. However, only a few studies have presented information about the association between SHBG and hypertension. In a prospective study in men, aimed to investigate the role of sex hormones in hypertension, Khaw et al. [5] observed that total testosterone but not SHBG was associated with hypertension. However, in accordance with our results, they found an inverse association between SHBG and diastolic blood pressure adjusted for age. The definition of hypertension in that study was different from our definition based on JNC7 (160/95 in Rancho Bernard and 140/90 in VSC). A cross-sectional study in men conducted in Tromsö [27], which aimed to investigate the association between testosterone on one hand and hypertension and left ventricular hypertrophy on the other hand, found an age-independent association between SHBG and both systolic and diastolic blood pressure. The association remained borderline significant for the systolic blood pressure (p=0,057) even after adjusting for BMI and alcohol consumption, but for diastolic blood pressure it was not significant (p=0,370). Significantly lower concentrations of SHBG were found in hypertensive men even after adjusting for age, BMI and SHBG concentrations in accordance with our results in women [27].

Some gender differences were, however observed in our study (Figure 1). In accordance with Khaw et al. [5] levels of SHBG decreased in a linear fashion in men when the blood pressure increased. In contrary in women the decrease in SHBG were firstly observed when they had developed hypertension. As the association between SHBG and hypertension in women was stronger over the age of 50 we suppose that these gender differences can at least in part be addressed to the menopause. In a prospective study including men and post-menopausal women, Ding et al. [17] observed a predictive value of SHBG concentrations concerning incident cases of type 2 diabetes, in both men and women. They reported an association between hypertension and low concentrations of SHBG in women in the crude analysis. However, the authors did not report whether any multivariate statistical tests accounting for possible confounders were computed. Neither was the association between SHBG and hypertension in males at baseline reported.

We did not found studies comparing the strength of the association between SHBG and hypertension with other risk factors for hypertension. In order to compare the SHBG with other conventional risk factors for hypertension, we standardized age, BMI, HOMA-IR and SHBG. In our study a weak association between insulin resistance and hypertension was observed in men were SHBG had stronger association with hypertension than HOMA-Ir.

Tot-T but not free testosterone was associated with hypertension in our population. Testosterone deficiency has been indicated as a risk factor for obesity and cardiovascular diseases. The question as to whether T has a direct effect on blood vessels remains to be investigated. We cannot confirm the vasodilatative effects of free testosterone that were shown by Webb et al. [8]. Our results suggest that the association between SHBG and blood pressure may explain the association between testosterone and blood pressure, at least partially. A recent study has shown that SHBG but not testosterone is associated with increasing of blood pressure [28]. In the matter of diabetes risk, Lakshman *et al*. [18] speculate on a modulatory effect of SHBG that enhances the effects of testosterone. In fact, a membrane receptor for SHBG is present in uterine endometrial cell membranes, isolated prostatic cell membranes, human placenta, normal breast, liver and epididymis but not in striated muscle [29]. This receptor, when activated after contact with SHBG, can stimulate the production of c-AMP. It must be emphasized that in a minor study with men with hypertension it was found a strong association between SHBG and renin that can be another pathway for direct effects of SHBG on the control of blood pressure [30]. Whether this association occurs in the blood vessel and if it influences the blood pressure remains to be confirmed by studies of a different design. For example, experimental studies on mice with knocked gene for SHBG could help in understanding the functions of the protein, and determine whether this protein only has carrying functions or if the functions of the protein are extended inside the target cells for androgen effect.

In accordance with previous studies, we found a positive linear association between SHBG and age in men while in women the association is inverse before 50 years of age [31,32]. The mechanisms lying behind this gender duality are not fully understood. Some authors suggest that in men a decrease of testosterone concentrations due to age is the cause of increasing SHBG concentrations as testosterone is supposed to have an inhibitory effect on SHBG production. Such an effect does not seem to be determinant in increasing SHBG concentrations in aging men according to De Ronde *et al.*[33].

### Strengths

This was a large population based study that included both men and women. Therefore, we could compare the association of oestradiol, SHBG and testosterone in both men and women. The study had a high participation rate of 76%, including a younger middle-aged population. The diagnosis of hypertension was précised with the trained nurse. In this large study population, we also had information about the medication influencing the blood pressure that gave us the possibility to exclude subjects with blood pressure lowering medication or with hormonal medication that could have influenced the analyses. Blood samples were collected in the morning, thereby avoiding influences of circadian changes in the hormonal concentrations.

### Limitations

A potential weakness of this study might be the lack of information with regard to menopausal status. Still, the use of 50 years as cut-off point for menopause is supported by findings from previous studies [34,35]. It should also be emphasized that as this is a cross-sectional study the causality between SHBG and hypertension cannot be established and it is possible that hypertension or associated factors lead to decreased concentrations of sex hormone-binding globulin. Another limitation of the study is the low accuracy of the measurements of testosterone in the low range of the distribution thus concerns especially women [36]. Moreover there is a circadian variation of concentrations of testosterone and SHBG which probably dilute our findings [37].

## Conclusion

These results show a strong association between SHBG and blood pressure independent of the components of metabolic syndrome and inflammation. This association might be explained by a direct effect of SHBG in endothelial cells through the receptor for SHBG. If others confirm this it might become a new field for the development of therapies for lowering blood pressure.

## Abbreviations

SHBG: Sex hormone-binding globulin; FAI: Free androgen index; JNC: Joint National Committee on Detection, Evaluation and Treatment of High Blood Pressure; BMI: Body mass index; HOMA: IR-homeostatic model assessment of insulin resistance; CRP: c-reactive protein; HDL: High density lipoprotein; LDL: Low density lipoprotein; Tg: Triglycerides; LTPA: Leisure time physical activity; OGTT: Oral glucose tolerance test; WHO: World Health Organisation; OR: Odds ratio; CI: Confidence interval; SD: Standard deviation.

## Competing interests

The authors report no competing interest. The authors alone are responsible for the content and writing of this paper.

## Authors’ contributions

BD prepared the data, performed the statistical analyses, drafted the manuscript and took part in conceiving the study. TR and PAJ offered their expertise in endocrinology and metabolism questions. CAL worked on preparing data and offered expertise in statistical analysis. LR conceived the study and acquired the data. UL conceived and coordinated the study, as well as acquired the data. All authors took part in the design of the study, the interpretation of data, the revision of the manuscript, and read and approved the final manuscript.

## Pre-publication history

The pre-publication history for this paper can be accessed here:

http://www.biomedcentral.com/1471-2261/13/30/prepub
